# Coronary artery aneurysm: case report

**DOI:** 10.1186/1749-8090-3-1

**Published:** 2008-01-24

**Authors:** Jeffrey E Everett, Harold M Burkhart

**Affiliations:** 1 Division of Cardiothoracic Surgery, University of Tennessee Medical Center, 1940 Alcoa Highway, Suite E-260, Knoxville, Tennessee 37920, USA; 2 Division of Cardiac Surgery, Mayo Clinic, 200 First Street SW, Rochester, MN 55905, USA

## Abstract

**Introduction:**

Aneurysms of the left main coronary artery are rare with an incidence of 0.1% in large angiographic series. The majority are atherosclerotic in origin. Other causes include connective tissue disorders, trauma, vasculitis, congenital, mycotic and idiopathic. The primary complication is myocardial ischemia or infarction, with rupture being rare. Treatment options include anticoagulation, custom made covered stents, reconstruction, resection, and exclusion with bypass.

**Case Presentation:**

A 66 year-old man was referred for evaluation of a 2 × 2 centimeter saccular aneurysm originating from the distal left main coronary artery. There was associated calcification and mild stenosis of the LM. The workup was prompted by a non-ST elevation myocardial infarction suffered following a laparotomy for a ruptured appendix. The past medical history was pertinent for hypertension, hyperlipidemia, and a left carotid endarterectomy.

Cardiopulmonary bypass with hyperkalemic cardioplegic arrest was utilized. The aneurysm was exposed in the atrioventricular groove. The aneurysm was resected and oversewn. Calcification precluded patch angioplasty. The patient then underwent coronary bypass grafting with the left internal thoracic artery placed to the left anterior descending artery and a reversed greater saphenous vein graft to an obtuse marginal branch of the circumflex artery. The postoperative course was uneventful and discharge to home occurred on the fourth postoperative day. Surgical pathology confirmed an atheromatous coronary artery aneurysm.

**Conclusion:**

Left main coronary artery aneurysms in adult patients are predominantly atherosclerotic in origin. The clinical presentation is that of myocardial ischemia, likely from associated embolism. Rupture is rare. Operative treatment is exclusion and revascularization.

## Introduction

Aneurysms of the left main (LM) coronary artery are rare with an incidence of 0.1% in large angiographic series [1]. The majority are atherosclerotic in origin. Other causes include connective tissue disorders, trauma, vasculitis, congenital, mycotic and idiopathic. Giant coronary aneurysm may be associated with fistulas to a cardiac chamber, most commonly the left ventricle. Most aneurysms are clinically silent, but those with an associated fistula may have an audible murmur and sign and symptoms of congestive heart failure. The primary complication is myocardial ischemia or infarction, likely secondary to embolism. Rupture of these aneurysms is rare. Treatment options include anticoagulation, custom made covered stents, reconstruction, resection, and exclusion with bypass [2-4]. The purpose of the case report is to highlight the clinical presentation, work-up and treatment options for a patient with a LM coronary artery aneurysm.

## Case presentation

**Figure 1:**
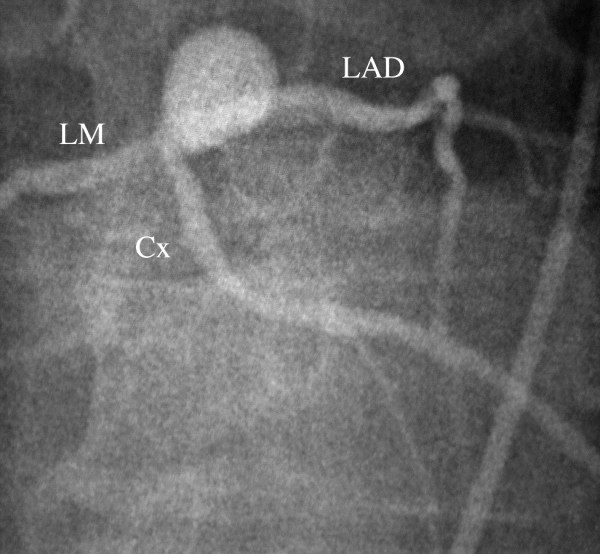
Coronary angiogram demonstrating the distal left main aneurysm.

A 66 year-old man was referred to our institution for evaluation of a 2 × 2 centimeter saccular aneurysm (Fig 1) originating form the distal left main coronary artery. The workup was prompted by a non-ST elevation myocardial infarction suffered following a laparotomy for a ruptured appendix. The patient's past medical history was pertinent for hypertension, hyperlipidemia, and a previous left carotid endarterectomy. Medications included a statin, beta blocker and aspirin. On physical exam the patient was mildly obese and free of chest pain. Review of the coronary angiogram revealed calcification and mild stenosis of the LM, in addition to the aneurysm. The left ventricular function was normal. The patient was anticoagulated with heparin. Cardiac enzymes normalized over several days.

The patient was prepared for surgery. Cardiopulmonary bypass with hyperkalemic cardioplegic arrest was utilized. The aneurysm was exposed in the atrioventricular groove by retracting the main pulmonary artery trunk rightward. The aneurysm was resected and oversewn. Calcification precluded patch angioplasty or resection with interposition graft placement. The patient then underwent coronary bypass grafting with the left internal thoracic artery anastamosed to the left anterior descending artery (LAD) and a reversed greater saphenous vein graft to an obtuse marginal branch of the circumflex (Cx) artery. The postoperative course was uneventful and discharge to home occurred on the fourth postoperative day. Surgical pathology confirmed an atheromatous coronary artery aneurysm.

## Conclusion

Coronary aneurysms are predominately atherosclerotic in origin in adult patients. They typically present with signs and symptoms of myocardial ischemia as noted in this case. This is attributed to embolism of atheromatous debris from with the aneurysm. Rupture of coronary aneurysms is rare and is not the indication for surgical intervention. Treatment to date has been based primarily upon anecdotal reports as no controlled trials of therapy exist. Anticoagulation has been the mainstay of nonoperative treatment. Percutaneously delivered custom made covered stents have been reported as another form of management. Surgical treatment is aimed at exclusion from the circulation either through resection or ligation with revascularization through bypass. Proximal aneurysms of the left main coronary artery may require aortotomy and patch closure of the orifice to gain proximal control [5]. Surgical repair can be performed with minimal morbidity and mortality.

## Competing interests

The author(s) declare that they have no competing interests.

## Authors' contributions

All authors read and approved the final manuscript.
